# Evaluation of epicardial adipose tissue in children with type 1 diabetes

**DOI:** 10.1038/s41390-024-03319-9

**Published:** 2024-06-15

**Authors:** Gül Trabzon, Şükrü Güngör, Şeyma Demiray Güllü, Osman Fırat Çalışkan, Ufuk Utku Güllü

**Affiliations:** 1 Hatay Mustafa Kemal University Medical Faculty Department of Pediatric Endocrinology, Hatay, Turkey; 2 Inonu University Medical Faculty Department of Pediatric Gastroenterology, Malatya, Turkey; 3 Hatay Mustafa Kemal University Medical Faculty Department of Pediatrics, Hatay, Turkey; 4 Hatay Mustafa Kemal University Medical Faculty Department of Pediatric Cardiology, Hatay, Turkey

## Abstract

**Introduction:**

Epicardial adipose tissue (EAT), the visceral fat surrounding the heart between the myocardium and visceral pericardium, intersects with Type 1 diabetes (T1D). This review aims to elucidate the intricate association between EAT and childhood T1D.

**Materials and methods:**

In this retrospective study, two pediatric groups were involved children with type 1 diabetes, and healthy children. Epicardial fat thickness was measured appropriately, and the study documented HbA1c levels and time to diabetes diagnosis for comprehensive analysis.

**Results:**

Encompassing 51 children with T1D and 69 healthy controls, revealed that children with type 1 diabetes had a mean HbA1c level of 9.4 ± 0.2, and a mean insulin dose of 0.94 units/kg/day. Epicardial adipose tissue (EAT) values were significantly higher in the Type 1 DM group. It has been shown that epicardial fat thickness may have a specific and sensitive value in type 1 diabetics.

**Discussion:**

The increased presence of epicardial fat tissue in children with type 1 diabetes is highlighted, prompting the consideration of various mechanisms. However, the complexity of this relationship underscores the need for further studies to provide a more comprehensive understanding of the underlying factors. Ongoing research in this area is crucial for advancing our knowledge and potential therapeutic interventions.

**Impacts:**

- Cardiac complications are one of the most important causes of morbidity and mortality in people with type 1 diabetes.
- Being able to detect cardiological complications of diabetes at an early stage contributes to morbidity.
- We found that epicardial fat tissue thickness was thicker in children with type 1 diabetes than in healthy children.
- Epicardial fat tissue thickness may be associated with poor control in children with type 1 diabetes and maybe a guide in terms of cardiac risks.

## Introduction

The visceral fat deposit surrounding the heart, known as epicardial adipose tissue (EAT), is located between the myocardium and the visceral pericardium. It consists of adipocytes, connective tissue, blood vessels, and immune cells. EAT is considered a biologically active tissue that secretes a variety of bioactive molecules such as adipokines, cytokines, and free fatty acids with the potential to affect both local and systemic functions. It exhibits a strong link to coronary artery disease and has been shown to play a role in the pathophysiology of This comprehensive review aims to elucidate the complex relationship between EAT and T1D in children by exploring the underlying mechanisms and clinical outcomes, metabolic disorders, inflammation, and cardiovascular diseases.^1,2^

In epicardial adipose tissue (EAT) assessment, echocardiography is extensively used due to its widespread availability and non-invasive nature. However, this method grapples with significant limitations, including variability between operators and within the same operator’s repeated measurements, alongside an inherent incapacity to accurately gauge EAT volume or delineate specific regional distributions of EAT, such as peri-atrial and pericoronary areas. Conversely, computed tomography (CT) offers distinct advantages in measuring both the volume and thickness of EAT, enabling precise localization of regional EAT positions (e.g., peri-atrial, peri coronary) and assessment of EAT density using Hounsfield units, in addition to quantifying pericardial adipose tissue (PAT) thickness and volume.^3,4^ Nevertheless, the minimally invasive nature of CT and the associated radiation exposure render it a less suitable option for pediatric populations. Given these considerations, magnetic resonance imaging (MRI) emerges as a superior alternative for pediatric EAT measurement, circumventing the radiation risk while providing a reliable and accurate assessment of EAT volume, thickness, and regional distribution without the limitations observed in echocardiography. Finally, MRI stands as the preferred modality for a more precise and safer evaluation of EAT in children, balancing the need for detailed anatomical resolution with patient safety concerns.^4^

Type 1 diabetes (T1D) is a challenging autoimmune disorder characterized by the destruction of pancreatic beta cells, leading to lifelong dependence on exogenous insulin.^5^ While extensive research has been conducted on the etiology and treatment of T1D, recent studies have revealed a potential association between T1D and epicardial adipose tissue (EAT) in the pediatric population.^6^

## Materials and methods

### Study design

This retrospective study was conducted by including two groups of pediatric patients. The first group (Group 1) consisted of 51 children diagnosed with type 1 diabetes mellitus who were under follow-up at the Pediatric Endocrinology Department of Hatay Mustafa Kemal University. The second group (Group 2) included 69 children aged 1-18 years without diabetes who were referred to the Pediatric Cardiology Department due to detected heart murmurs during sports license evaluations, without any identified cardiac pathology. Inclusion criteria for the diabetic group involved patients with type 1 diabetes without additional comorbidities who either had heart murmurs during an examination or were referred to pediatric cardiology due to symptoms such as palpitations or chest pain.

The work was evaluated and approved by the Ethnic Committee of the University of Hatay Mustafa Kemal. All procedures performed in studies involving human participants were by the ethical standards of the institutional and/or national research committee and with the 1964 Helsinki Declaration and its later amendments or comparable ethical standards.

### Participant selection

Participants were divided into two groups based on their medical conditions. Group 1 included children with type 1 diabetes, while Group 2 served as the control group of healthy children without diabetes. Patients in both groups were matched for age, sex, and anthropometric measurements. All participants had normal blood pressure and lipid levels. The insulin doses taken by children with type 1 diabetes were similar according to weight, and none of them had an insulin pump and were using more than one insulin.

### Data collection

Height, weight, and waist circumference measurements were taken at the Pediatric Endocrinology Clinic. Body mass index (BMI) was calculated for each participant. Additionally, echocardiographic measurements were performed for all patients at the Pediatric Cardiology Clinic. During echocardiographic assessments, the HbA1c levels at the time of measurement and the duration of diabetes diagnosis were noted.

### Echocardiographic measurement of epicardial adipose tissue

A single pediatric endocrinologist made echocardiographic measurements. Each participant received a transthoracic two-dimensional guided M-mode echocardiogram. The ECHO procedure was performed with the Philips iE33 xMATRIX ultrasound device according to the pediatric ECHO guide of the American Society of Echocardiography. Epicardial fat tissue thickness was measured perpendicular to the myocardial wall in the parasternal long axis in the lateral decubitus position.^7^ During end-diastole, measurements were taken, as these values exhibit greater consistency with cardiac magnetic resonance imaging.^8,9^ A total of 3 measurements were taken and the average was taken.

### Statistical analysis

Counts and percentages were used to summarize demographic and baseline clinical factors for each group with categorical variables, while mean ± SD was employed for continuous variables. Pearson correlations were utilized to explore the relationships between EAT, clinical characteristics, and cardiovascular measurements. ROC analysis was performed to evaluate the correct classification of epicardial fat tissue to determine the best cut-off point and to evaluate sensitivity and specificity values at different cut-off points. The optimal cut-off point was determined to achieve maximum sensitivity and specificity.

Demographic and baseline clinical factors were summarized for each group using counts and percent for categorical variables and mean ± SD for continuous variables. Associations between EAT, clinical characteristics, and cardiovascular measurements were examined using Pearson correlations. Differences in EAT between groups were compared by independent sample *t* tests and a general linear model adjusting for age, sex, and BMI. Multivariate linear regression analysis adjusting for age, sex, BMI, and diabetes status was used to examine EAT as a predictor for measurements of cardiovascular function that showed significant bivariate correlation. Data were analyzed using PASW Statistics 18 (PASW Statistics for Windows, Version 18.0. Chicago: SPSS Inc.) with significance set at *p* < 0.05. Cohen’s d was used as a measure of effect size for group comparisons.

This study aimed to compare the cardiac health and anthropometric measurements of children with type 1 diabetes to those of healthy children and to evaluate the relationship between these factors and diabetes-related parameters.

## Results

A total of 120 children, 51 with type 1 diabetes and 69 healthy controls, were enrolled. 48% of the children were female and 62% were male and there was no statistical difference between the two groups. The mean age was 10.67 ± 4.05 years in children with type 1 diabetes and 10.65 ± 3.76 years in healthy controls, with no statistically significant difference between the two groups. The mean BMI z score of children with type 1 diabetes was –0.242 ± 1.550, the mean BMI z score of healthy controls was—0.395 ± 0.967 and there was no statistically significant difference between the two groups in terms of VKI index. There was no statistically significant difference in terms of age, sex, weight Z-score and height Z-score, or BMI Z-score in the patient and control groups included in the study (p:0.970, p:0.897, p:0.696, p:0.585, p:0.508, respectively). The mean HbA1c level of children with type 1 diabetes was 9.4 ± 0.2, the mean duration of diabetes was 29.4 ± 4.4 months and the mean insulin dose was 0,94 ü/kg/day. When echocardiography findings were evaluated; EF and pulmonary flow were normal in both groups and there was no statistically significant difference between the groups but EAT values were found to be statistically significantly higher in the Type 1 DM patient group (*p* < 0.001) (Table 1).

**Table 1:** Evaluation of differences in demographic and cardiac findings between groups.

		Control (69)	Type 1 diabetes (51)	*P*
		n-%	n-%	
Sex	female	33-47.8	25-49	0.897*
	Male	36-52.2	26-51	
		Mean ± SD	Mean ± SD	
Age in years		10.65 ± 3.76	10.67 ± 4.05	0.970
Weight Z score		−0.271 ± 0.894	−0.189 ± 1.287	0.696
Height Z score		0.002 ± 1.038	−0.128 ± 1.587	0.585
VKİ Z score		−0.395 ± 0.967	−0.242 ± 1.550	0.508
EF		72.72 ± 3.37	73.96 ± 3.85	0.065
KF		41.43 ± 3.19	42.35 ± 3.53	0.139
Pulmonary flow		1.051 ± 0.065	1.059 ± 0.113	0.640
EAT(epicardial adipose tissue)		0.426 ± 0.411	0.861 ± 0.287	<0.001

Statistics:

*Crosstabs Chi-square test.

Independent student *T* test.

When the direction of the relationship between clinical and laboratory findings and ECO findings in patients with Type 1 DM was evaluated, there was no significant correlation between waist circumference, glucose levels, duration of diabetes, and EAT. There was a moderately significant positive correlation between HbA1c and EAT, (r: 0.323 p: 0.027, respectively). There was a moderate positive correlation between BMI Z score and EAT, (r: 0.205 p: 0.006, respectively) (Table 2). In the ROC curve analysis we conducted to determine the best cut-off point of EAT thickness for Type 1 DM, we found that EAT thickness of 0.3 mm and above had 100% sensitivity and 65.2% specificity for Type 1 DM (*p* < 0.001)(Table 3).

**Table 2:** Evaluation of the relationship between clinical and laboratory findings and epicardial adipose tissue(EAT) in children with type 1 diabetes

		EAT
Waist circumference	Pearson Correlation	0249
	Sig. (2-tailed)	0221
Duration of diabetes	Pearson Correlation	0122
	Sig. (2-tailed)	0393
Glucose	Pearson Correlation	0069
	Sig. (2-tailed)	0668
HbA1c	Pearson Correlation	0323^*^
	Sig. (2-tailed)	0027
BMI Z score	Pearson Correlation	0250
	Sig. (2-tailed)	0006

**Table 3:** Tip 1 Determining the best EAT interval for DM

	Best cut-off point for type 1 DM	Sensitivity	Specificity	AUC	%95 CI	*P*
**EAT (mm)**	0.3	0.100	0.652	0.808	0.727-0.889	<0.001

Statistics: ROC Curve analysis.

*AUC* area under curve, *CI* confidence interval.

## Discussion

In our study, we observed elevated levels of EAT in children with T1D when compared to age, sex, and BMI-matched healthy counterparts. Although studies on this subject in children are limited, the findings were consistent with the studies conducted by Güney et al. and Chambers et al.^1,2^

Although the reason for the increase in epicardial adipose tissue in patients with type 1 diabetes is not fully understood, publications are indicating that this may be related to the insulin dose used, and if the dose used is high or the hba1c is low, this may be related to the high insulin dose.^10,11^ On the other hand, it is known that although fat stores are reduced in insulin deficiency, epicardial adipose tissue is protected from this reduction.^5^ The insulin doses used in our patients were similar and not high (mean 0.94 ü/kg/day) and HbA1c levels were high (mean 9.4%) in contrast to low levels. There was also a positive correlation between hba1c level and epicardial adipose tissue. This result showed that different mechanisms other than the insulin used are also effective in the increase of epicardial adipose tissue in type 1 diabetics. This may be secondary to oxidative stress and increased inflammation caused by increased blood glucose in insulin deficiency and the inability of heart cells to utilize enough glucose.^6^ The increase in epicardial adipose tissue as hba1c increases supports this hypothesis. The lack of a correlation between glucose levels and EAT is attributed to the fact that instantaneous glucose levels in individuals with type 1 diabetes can be easily affected by diet, exercise, and insulin dosage. This observation underscores the complex interplay between metabolic regulation and lifestyle factors in managing this condition.

In contrast to previous studies, the lack of a correlation between waist circumference and EAT may be attributed to the small number of patients whose waist circumference was measured because the study was retrospective.^12^ This is also a limitation of our study. The positive correlation between BMI and epicardial adipose tissue in patients with type 1 diabetes, while there is no correlation in the healthy control group; suggests that different pathophysiologies contribute to the increase in epicardial adipose tissue other than the increase in visceral adipose tissue. This situation emphasizes the need for comprehensive randomized controlled prospective studies.

The epicardial adipose tissue of 0.3 mm, which we found in children with type 1 diabetes as a result of our analysis in this study, can be used in the evaluation of treatment compliance of children with type 1 diabetes with thicknesses above this value and the control of glucose regulation. In addition, it should be known that the risk of coronary artery disease increases in diabetics with increased epicardial fat tissue thickness, and patients should be followed in this regard and strict precautions should be taken in this regard.

## Conclusion

In conclusion, epicardial adipose tissue was increased in children with type 1 diabetes compared to similar healthy subjects according to age, sex, and BMI. The increase in EAT thickness with increasing HbA1c level indicates that poor control increases this thickness. Values of 0.3 mm and above may guide us about poor control. Epicardial fat tissue thickness may be associated with poor control in children with type 1 diabetes and may be a guide in terms of cardiac risks.

Our manuscript incorporates a dedicated section emphasizing the imperative for prospective, longitudinal studies to unravel the long-term cardiovascular impacts associated with variations in epicardial adipose tissue (EAT) thickness in children with Type 1 Diabetes (T1D). Our preliminary findings underscore the necessity of broadening our investigation to explore how subtle changes in EAT thickness may serve as indicators of cardiovascular risk over time. Conducting such comprehensive research is crucial for developing a deeper understanding of EAT’s prognostic significance in the pediatric T1D population. Additionally, assessing the variability of EAT measurements across different demographic segments—including age, sex, and ethnicity—is vital for ensuring the robustness and applicability of our conclusions. By doing so, we aim not only to affirm the clinical significance of the 0.3 mm EAT thickness threshold but also to enhance our understanding of the complex pathophysiological and epidemiological factors at play in T1D, thereby informing more effective intervention and risk mitigation strategies.

## Data Availability

The data used in this study, pertaining to epicardial adipose tissue in individuals with Type 1 diabetes, is not publicly available due to privacy and confidentiality concerns. However, researchers interested in replicating or validating our findings may request access to the anonymized dataset by contacting the corresponding author. Access to the data will be provided in accordance with ethical and legal standards, ensuring participant confidentiality and privacy protection. Requests for data access will be reviewed by the research team and the Hatay Mustafa Kemal University Ethics Committee documents on 26.07.2023 with decision number 17 to ensure compliance with relevant regulations.
